# Divergent DNA methylation patterns associated with gene expression in rice cultivars with contrasting drought and salinity stress response

**DOI:** 10.1038/srep14922

**Published:** 2015-10-09

**Authors:** Rohini Garg, VVS Narayana Chevala, Rama Shankar, Mukesh Jain

**Affiliations:** 1 Functional and Applied Genomics Laboratory, National Institute of Plant Genome Research (NIPGR), Aruna Asaf Ali Marg, New Delhi - 110067, India

## Abstract

DNA methylation is an epigenetic mechanism that play an important role in gene regulation in response to environmental conditions. The understanding of DNA methylation at the whole genome level can provide insights into the regulatory mechanisms underlying abiotic stress response/adaptation. We report DNA methylation patterns and their influence on transcription in three rice (*Oryza sativa*) cultivars (IR64, stress-sensitive; Nagina 22, drought-tolerant; Pokkali, salinity-tolerant) via an integrated analysis of whole genome bisulphite sequencing and RNA sequencing. We discovered extensive DNA methylation at single-base resolution in rice cultivars, identified the sequence context and extent of methylation at each site. Overall, methylation levels were significantly different in the three rice cultivars. Numerous differentially methylated regions (DMRs) among different cultivars were identified and many of which were associated with differential expression of genes important for abiotic stress response. Transposon-associated DMRs were found coupled to the transcript abundance of nearby protein-coding gene(s). Small RNA (smRNA) abundance was found to be positively correlated with hypermethylated regions. These results provide insights into interplay among DNA methylation, gene expression and smRNA abundance, and suggest a role in abiotic stress adaptation in rice.

Epigenetic modifications play a crucial role in the regulation of chromatin structure and modulate gene activity in eukaryotes. DNA methylation at cytosine residues and post-translational histone modifications are the major epigenetic modifications. DNA methylation is a stable and heritable modification present throughout the genome and is crucial for maintenance and coordination of various cellular processes and developmental programs. It has been reported that variation in DNA methylation could be responsible for heritable phenotypic differences, including agronomically important traits in plants^1^,^2^,^3^.

In plants, DNA methylation is present in three different sequence contexts, CG, CHG and CHH (where H = A, C or T). Cytosine methylation is established and maintained by *de novo* methyltransferases (DRM1/2/CMT3) via RNA-directed DNA methylation (RdDM) pathway and MET1 proteins^4^,^5^. DNA methylation has been proposed as one of the major factors that can modulate gene expression. It has been demonstrated that DNA methylation inhibits the transcription and movement of transposons for protection of the genome^6^,^7^. The methylation of protein-coding genes and transposons can also affect the transcription of neighbouring genes^8^,^9^,^10^. Several studies have demonstrated the crucial role of DNA methylation in diverse plant developmental processes, such as seed/embryo development and gametophyte development^7^,^11^,^12^,^13^,^14^. Although the role of DNA methylation has been well established in development, a few recent studies have suggested its role in stress responses as well^15^,^16^,^17^,^18^. However, most of research on DNA methylation-regulated abiotic stress responses has focused on few/specific loci based on low-throughput technologies^15^,^19^,^20^,^21^.

Rice is an important crop accounting for food security of over half the world population. Water-deficit and salinity are the major abiotic factors that affect rice productivity worldwide. Rice germplasm exhibit variability in their response to these abiotic stresses; some genotypes possess ability to tolerate extreme drought and salinity stresses, whereas many of them are highly susceptible. These phenotypic variability may be attributed to genetic and epigenetic variations, and different regulatory architecture among them. The study of tolerance/response mechanisms to abiotic stresses has been intensively worked out based on genomics, transcriptomics and proteomics analyses. Several genes and genomic variations involved in stress responses have been identified and a few regulatory networks have been proposed^22^,^23^,^24^,^25^. However, only a few studies have analyzed the epigenetic regulation of stress response in rice, that too at low-throughput level, mostly focused on a set of genes/loci^26^,^27^. High-throughput sequencing technologies provide an opportunity to recognize the DNA methylation at single-base resolution quickly and comprehensively in different biological contexts in plants^18^,^28^,^29^,^30^. Although several techniques have been applied to study genome-wide DNA methylation, most detailed methylome maps at single-base resolution have been obtained using bisulphite sequencing^31^,^32^,^33^,^34^.

A global analysis of extent and pattern of DNA methylation in rice varieties with contrasting response to abiotic stresses is lacking. In this study, we investigated the differences in DNA methylation patterns of three different rice cultivars with contrasting responses to drought and salinity stresses. The expression of stress-responsive genes was found to be influenced by methylation status of associated regions, which suggested possible role of DNA methylation in stress adaptation. The high resolution methylome maps of different rice genotypes and differentially methylated regions contributes to our understanding of the role of epigenetic regulation of stress responses in plants.

## Results

Three well-characterized rice cultivars [IR64, drought and salinity sensitive; Nagina22 (N22), drought-tolerant; Pokkali, salinity-tolerant] with contrasting response to drought and/or salinity stresses, were used in this study for genome-wide analysis of DNA methylation, gene expression and small RNA profiling to reveal the epigenetic regulation of abiotic stress response. These rice cultivars are commonly used worldwide for the generation of various genomic resources and donors of stress-related agronomic traits^22^,^25^,^35^,^36^.

### DNA methylation landscape of rice genome in different cultivars

**Figure 1:**
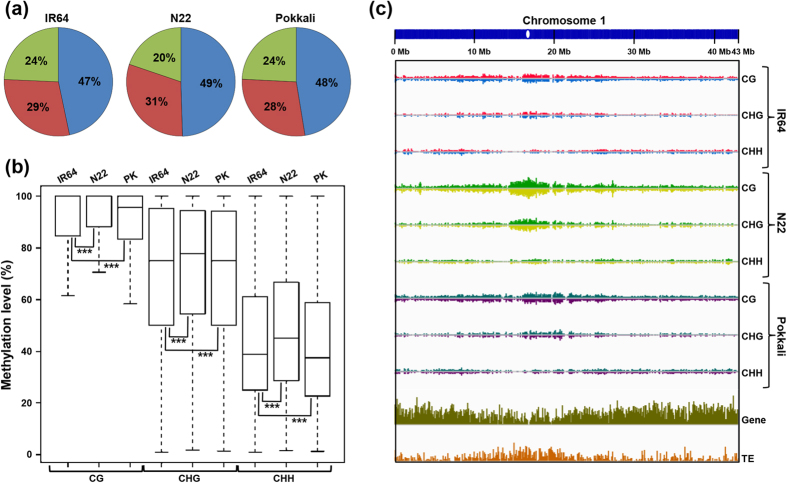
DNA methylation status and chromosomal distribution in different rice cultivars. (**a**) The relative fraction of methylcytosines (mCs) identified in each sequence context (CG, CHG and CHH) in three rice cultivars, IR64, N22 and Pokkali. Blue, CG; maroon, CHG; green, CHH. (**b**) Box-plot showing distribution of DNA methylation level in each sequence context in different cultivars. Significant differences (Fisher’s exact text) are marked with asterisks (***P < 1 × 10^−10^). (**c**) The density of mCs (identified in each sequence context on two DNA strands), genes and transposable elements (TEs) throughout chromosome 1 (bin size 100 kb). N22, Nagina 22; PK, Pokkali.

Genome-wide profiling of DNA methylation using bisulphite sequencing was performed in IR64, N22 and Pokkali rice cultivars. In total, 160–212 million high-quality reads were obtained (Supplementary Table S1), reflecting >30 × genome coverage for each sample. Only the sequence reads that mapped uniquely to the reference rice genome were considered for the analysis. The uniquely mapped reads provided 87–89% coverage of the rice genome and over 80% (81–83%) of cytosines (Cs) in the genome (Supplementary Table S1). Using the non-conversion rate of Cs (0.05%) in the non-methylated chloroplast genome as error rate, the methylation status of each C was determined at a *P*-value cut-off of 0.001. Applying this criteria, we identified methylated C residues (mCs) for which sufficient read depth (at least five) was available. Overall, the frequencies of mCs were similar in IR64 (11.32%) and Pokkali (11.5%), and marginally higher in N22 (12.30%). Of the total mCs, the highest fraction was of CG (47–49%) followed by CHG (28–31%) and CHH (20–24%) for all the three rice cultivars (Fig. 1a). The methylation level of mCs in CG context was much higher than CHG and CHH contexts (Fig. 1b). The average methylation levels was also highest in CG context (87–88%) followed by CHG (67–68%) and CHH (41–43%) contexts (Supplementary Fig. S1). The relative frequency of mCs in different contexts and the tendency of higher methylation level in CG context and lower methylation levels in CHH context were similar to those observed in other plants^10^,^32^,^37^. A higher methylation was observed in N22 for Cs in all contexts (Fig. 1b). Overall, significant differences were observed in the methylation levels among different rice cultivars.

We observed extensive DNA methylation in the pericentromeric regions of the chromosomes as compared to ends, reflecting dense methylation of repetitive sequences (Fig. 1c, Supplementary Fig. S2). A considerable number of mCs were observed in the non-repetitive intergenic and genic regions as well. The comparison of DNA methylation levels with density of genes and transposable elements (TEs) revealed a positive correlation with the density of TEs and negative correlation with gene density. We found substantial variations between IR64 and N22 in the distribution of mCs in all contexts, whereas only few regions showed variations in distribution of mCs between IR64 and Pokkali (Fig. 1c, Supplementary Fig. S2). Further, we examined strand-specific distribution of DNA methylation in the three rice cultivars. Both DNA strands (sense and anti-sense) of the genome exhibited similar methylation levels with minor differences (Supplementary Fig. S3).

### DNA methylation patterns in protein-coding genes and TEs

**Figure 2:**
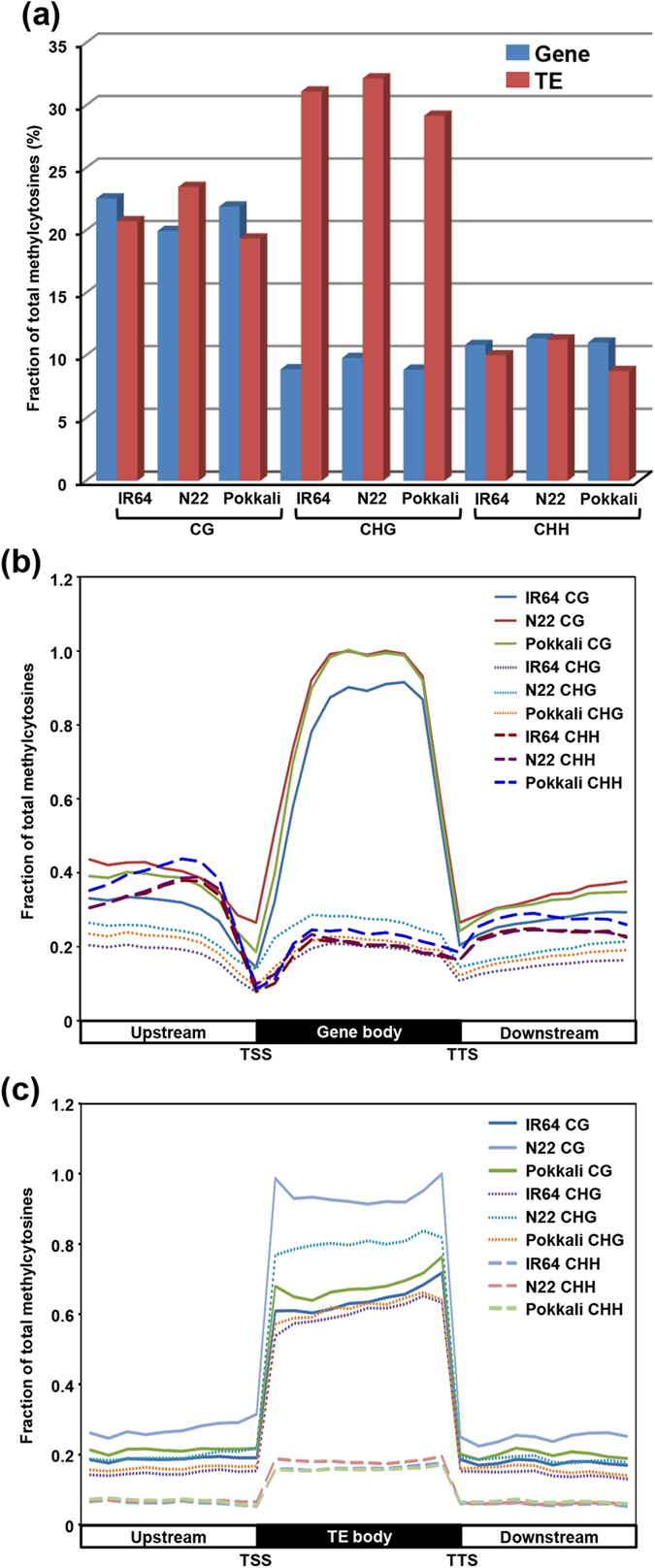
DNA methylation patterns in gene and transposable element (TE) regions in different rice cultivars. (**a**) Fraction of methylcytosines (mCs) identified in the body of protein-coding genes and TEs in each sequence context in rice cultivars. (**b**) Relative fraction of mCs within gene body and 1 kb flanking (upstream and downstream) sequences in each sequence context in different cultivars. (**c**) Relative fraction of mCs within TE body and 1 kb flanking (upstream and downstream) sequences in each sequence context in rice cultivars. TSS, transcriptional start site and TTS, transcriptional termination site.

We analyzed DNA methylation patterns in genic and TE regions. In general, CHG methylation levels were much higher in TEs (28–32%) as compared to protein-coding genes (7–9%) in all the rice cultivars (Fig. 2a). The CG context methylation of TEs was also higher than genes in N22, which was in contrast to that of IR64 and Pokkali. In CHH context, the methylation level of TEs and genes was similar in N22, but was lower for TEs than genes in IR64 and Pokkali. These results suggest that protein-coding genes and TEs are differentially recognized for methylation in different rice cultivars. Further, we investigated patterns of DNA methylation in various genomic features in all contexts (Supplementary Fig. S4). For CG, largest number of mCs were located within gene body in rice cultivars. However, the frequency of mCs in CHG and CHH contexts were considerably higher in the upstream and downstream regions as compared to the gene body. Least number of CHH context mCs existed in the exonic regions (CDS and UTRs). Further, we analyzed the pattern of DNA methylation within gene body, and upstream and downstream flanking sequences. DNA methylation sites in all contexts increased rapidly while moving upstream to transcriptional start sites (TSSs) and downstream to termination sites (TTSs, Fig. 2b). In contrast to CHG and CHH, number of mCs in CG context was higher in the gene body as compared to the flanking sequences. Notably, the frequency of mCs in CHH context was higher than CG context mCs in the upstream region near the TSSs. Similar observations have been made in other plant species too^38^,^39^. However, the methylation level throughout the upstream, gene body and downstream regions was highest in CG context followed by CHG and CHH (Supplementary Fig. S5).

Because DNA methylation has a major role in transposon silencing, we examined the patterns of DNA methylation in TEs as well (Fig. 2c). The analysis revealed enrichment of DNA methylation throughout the TE body as compared to flanking sequences in all contexts. However, the enrichment of CG and CHG methylation was several-fold higher than CHH methylation. Notably, the methylation level was markedly higher in the TE body as compared to protein-coding genes. Also, TEs were highly methylated near the TSSs and TTSs as opposed to the protein-coding genes (Fig. 2b,c), consistent with earlier reports^10^,^13^. Among the rice cultivars, the patterns of DNA methylation in CHG and CHH contexts were almost similar, whereas substantial differences were observed for CG context. The TE body was most highly methylated in N22 followed by IR64 and Pokkali.

### Differentially methylated regions in different rice cultivars

**Figure 3:**
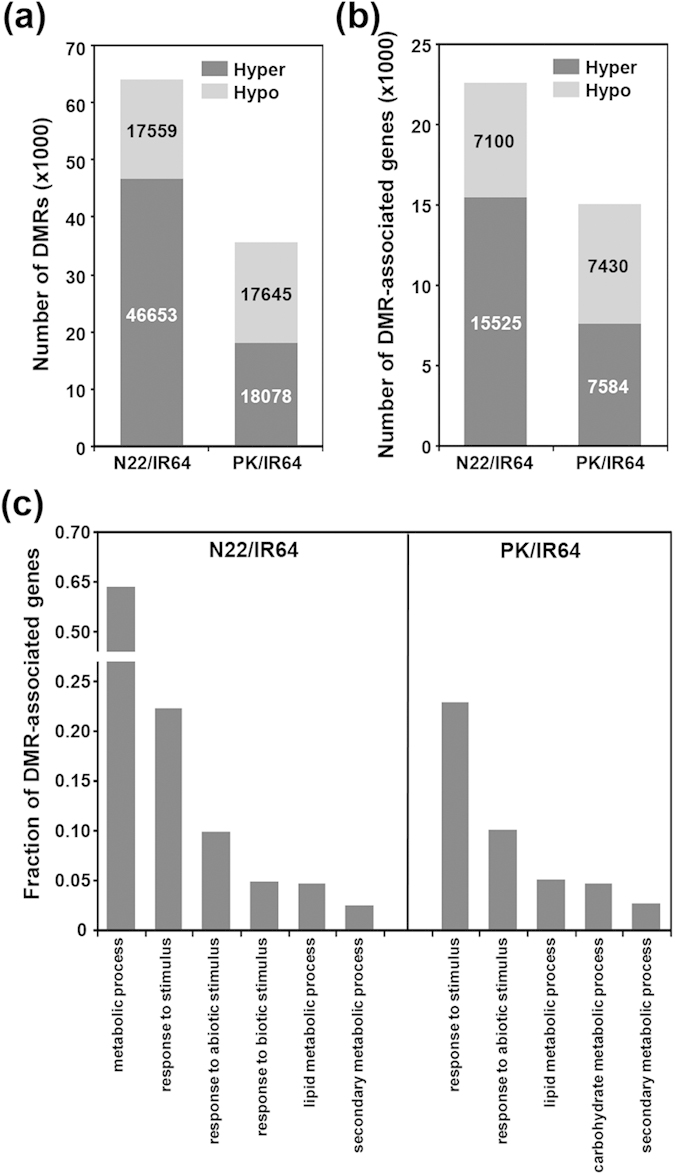
Differential methylation in rice cultivars. (**a**) Number of differentially (hyper and hypo) methylated regions (DMRs) between N22 and IR64 (N22/IR64) and Pokkali and IR64 (PK/IR64). (**b**) Number of protein-coding genes associated with DMRs. DMRs present within gene body or 2 kb flanking sequences were regarded as DMR-associated genes. (**c**) Gene ontology (GO) categories (biological process) significantly (*P*-value ≤ 0.05) enriched in the DMR-associated genes among rice cultivars. N22, Nagina 22;; PK, Pokkali.

To investigate the differential methylation in the three rice cultivars, we identified differentially methylated regions (DMRs). For identification of DMRs, we calculated methylation levels in 100 bp bins. A total of 64,212 DMRs between N22 and IR64 (N22/IR64), and 35,723 DMRs between Pokkali and IR64 (PK/IR64) could be identified (q-value ≤ 0.01, Fisher’s exact test followed by SLIM correction) (Fig. 3a). We observed that hypermethylation was more common in N22, whereas the frequency of hyper and hypomethylation was similar in Pokkali. The DMRs were found more likely to be located near (2 kb upstream and downstream) the genes (42–45%), but not so often within the gene body (16–18%) (Supplementary Fig. S6). Overall, the DMRs present within/near protein-coding genes presented the major fraction (57–62%) of total DMRs. The genes with DMRs within their body and 2 kb flanking sequences were regarded as DMR-associated genes. A large number of protein-coding rice genes were identified as DMR-associated genes in N22/IR64 (57.9%) and PK/IR64 (38.5%). In N22/IR64, number of hyper DMR-associated genes were significantly higher (2.2 times) than hypo DMR-associated genes, whereas the number of hyper and hypo DMR-associated genes were almost similar in PK/IR64 (Fig. 3b). Gene ontology (GO) analysis revealed that genes involved in diverse biological processes, such as metabolic processes, response to stress, signal transduction, translation and epigenetic regulation of gene expression were differentially methylated among rice cultivars (Supplementary Fig. S7). Notably, a large fraction of these genes were found to be associated with response to abiotic stress. The enrichment analysis of DMR-associated genes revealed significant overrepresentation of genes involved in metabolic processes (lipid metabolic process and secondary metabolic process) and response to stress in both N22/IR64 and PK/IR64 (Fig. 3c).

### Differential gene expression in different cultivars

**Figure 4:**
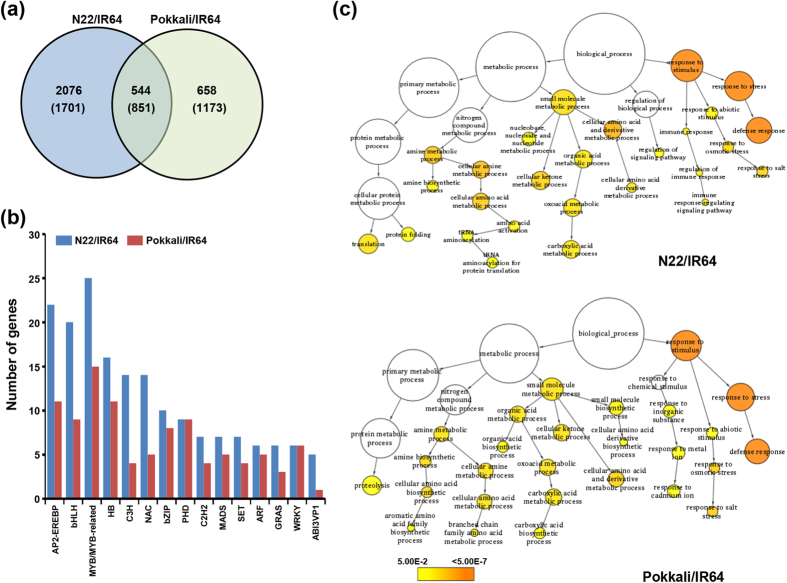
Differential gene expression among the rice cultivars and gene ontology (GO) enrichment analysis. (**a**) Venn diagram showing number of differentially expressed genes in N22/IR64 and Pokkali/IR64. Number of upregulated and downregulated (in parentheses) genes are shown. (**b**) Number of genes from top 15 transcription factor families represented in the differentially expressed genes in N22/IR64 and Pokkali/IR64. (**c**) GO enrichment analysis of genes showing differential expression in N22/IR64 and Pokkali/IR64. The significantly enriched GO terms are highlighted in different colours as per scale (*P*-value) given at the bottom.

We determined the transcript abundance of all the rice genes in IR64, N22 and Pokkali cultivars using RNA-seq approach (Supplementary Table S2). The transcripts showing at least two-fold change with *P*-value less than 0.05 were identified as differentially expressed genes (DEGs). A total of 5172 (2620 upregulated and 2552 downregulated) and 3226 (1202 upregulated and 2024 downregulated) rice transcripts were found to be differentially expressed in N22/IR64 and PK/IR64, respectively (Fig. 4a, Supplementary Table S3). Among these, 1395 (544 upregulated and 851 downregulated) transcripts were differentially expressed in both the comparisons. The genes involved in various cellular processes, including metabolic processes, amino acid metabolism, cell wall components, response to abiotic stimulus (osmotic stress, salt stress and water stress), defense response, photosynthesis, transcription and signal transduction were well represented among the differentially expressed genes (Supplementary Table S3). At least 356 transcription factor encoding genes belonging to 50 families were found to be differentially expressed in N22 and/or Pokkali as compared to IR64. The genes belonging to MYB/MYB-related, AP2-EREBP, bHLH and homeobox families were most highly represented among the differentially expressed genes (Fig. 4b). In addition, a large number of members of C3H, NAC, bZIP, PHD and WRKY families were also differentially expressed in N22 and/or Pokkali. GO enrichment analysis revealed that the genes involved in response to abiotic stimulus and amine metabolic processes were significantly enriched in both N22 and Pokkali cultivars (Fig. 4c). In addition, the genes involved in translation, protein folding and regulation of catalytic activity were significantly enriched in N22 cultivar. The changes in expression of many of these genes under stress conditions have been reported in previous studies too and implicated in stress tolerance mechanisms^10^,^35^,^36^,^40^,^41^. The modulation of various metabolic pathways, such as osmoprotectants, cell wall components, amino acid metabolism, lipid metabolism, hormone metabolism and secondary metabolites have been correlated with the physiological differences among the rice cultivars under stress conditions.

### Differential methylation is coupled to differential gene expression in different cultivars

**Figure 5:**
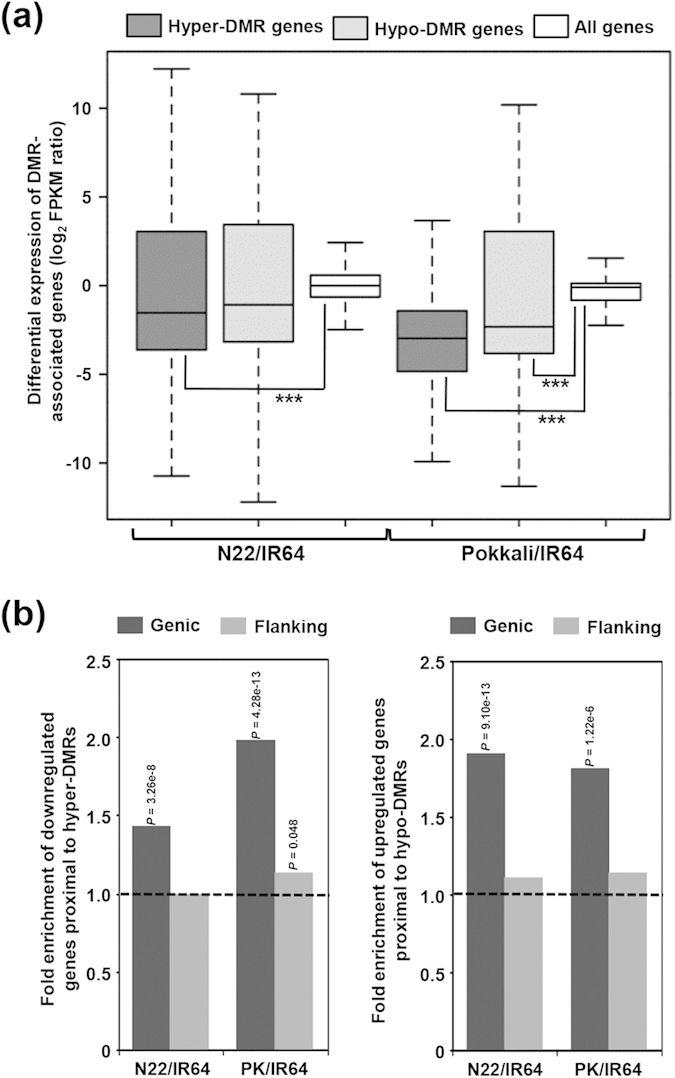
Relationship between differential methylation and transcript abundance of protein-coding genes. (**a**) Box-plot showing differential expression levels of all the genes or genes associated with hyper/hypo methylated DMRs are displayed. Significant differences (Fisher’s exact text) are marked with asterisks (***P < 1 × 10^−10^). (**b**) Enrichment of downregulated and upregulated genes proximal to hypermethylated and hypomethylated DMRs, respectively, positioned within the protein-coding genes (genic DMRs) or flanking (flanking DMRs) sequences. Significance (*P*-value by Fisher’s exact test) of the enrichment has been given. N22, Nagina 22; PK, Pokkali.

To examine influence of DNA methylation on expression of neighbouring genes, we assessed the relationship between DMRs and transcript abundance on a genome-wide scale. A total of about 39% (2010) and 30% (966) of the DEGs in N22/IR64 and PK/IR64, respectively, were found to be associated with DMRs. We found that differential expression of DMR-associated genes was dependent on the direction of change in methylation status (Fig. 5a). In general, the genes proximal to hypermethylated DMRs exhibited lower levels of transcript abundance (downregulation) relative to entire gene set. The genes proximal to hypomethylated DMRs displayed similar or moderately higher levels of transcript abundance (upregulation) compared to all genes. However, some of the DMR-associated genes showed positive correlation with the transcript abundance. DNA methylation appeared to be correlated with on/off status of gene expression too for DMRs (14.5% for N22/IR64 and 7.2% for PK/IR64) (Supplementary Fig. S8a,b). For other cases, DNA methylation state was correlated with quantitative differences in gene expression. DMRs with negative correlation with gene expression were found more likely located near genes boundaries opposed to those not associated with gene expression.

Furthermore, enrichment analysis of DMR-associated DEGs revealed a negative correlation between methylation status and transcript abundance (Fig. 5b). A significant enrichment of downregulated genes was observed in genes proximal to hyper-DMRs present within the gene body and vice-versa for both N22/IR64 and PK/IR64. However, no significant correlation was observed for the DMRs present in the flanking sequences except for PK/IR64. These results suggest that the gene body methylation play an important role in regulation of gene expression and might contribute, in large part, to the differential stress response of the rice cultivars.

### Differential methylation of genes associated with stress response and epigenetic regulation of gene expression

**Figure 6:**
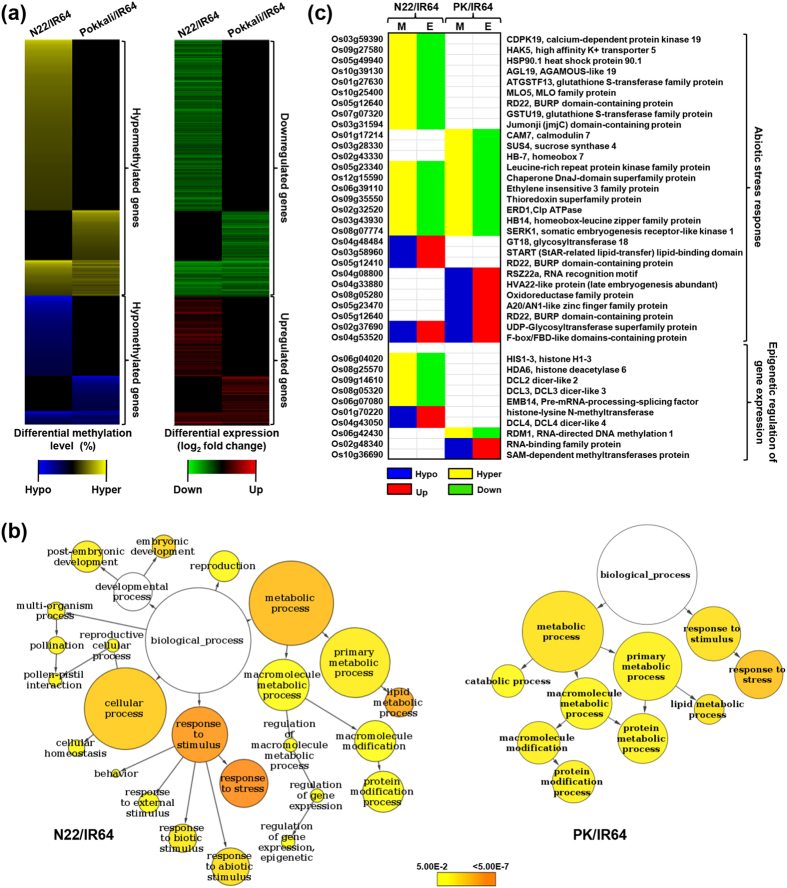
Differential methylation, transcript abundance and gene ontology enrichment of protein-coding genes. (**a**) Heatmap representation of the differential methylation levels and differential expression of DMR-associated genes showing negative correlation. Color scales at the bottom represent status of methylation and transcript abundance. (**b**) GO enrichment analysis of DMR-associated genes showing differential expression. The significantly enriched GO terms are highlighted in different colours as per scale (*P*-value) given at the bottom. (**c**) Heatmap representation of the differential methylation (M) and differential expression (E) of DMR-associated genes known to be involved in abiotic stress response and epigenetic regulation of gene expression. Gene identifiers (MSU v7) and gene descriptions (best Arabidopsis ortholog) are given on the left and right sides of the heatmap, respectively. Colors at the bottom represents status of differential methylation (hypo/hyper) and differential expression (up/down). N22, Nagina 22; PK, Pokkali.

The expression of genes involved in diverse cellular processes vary in different cultivars. The activation or repression of many of these genes is dependent on their chromatin structure, which is mainly determined by epigenetic marks, such as DNA methylation. Several examples of epigenetic regulation of gene expression in response to environmental stress are known^10^,^15^,^18^,^20^,^26^,^27^,^42^. We also found the differential methylation of several genes belonging to various functional categories in the rice cultivars. These genes encoded proteins involved in various cellular processes, such as transcription regulation, metabolic processes and signal transduction (Supplementary Table S4). A substantial number of genes encoded for proteins with unknown function. The differential methylation level and corresponding differential gene expression of rice genes in N22/IR64 and PK/IR64 are shown in Fig. 6a. The GO analysis of DMR-associated genes in both N22/IR64 and PK/IR64 revealed a significant enrichment of genes that participate in stress response (Fig. 6b,c). In addition, genes involved in metabolic processes, such as lipid metabolic processes were enriched in both the comparisons. The epigenetic regulation of gene expression and protein modification processes were other important GO terms significantly overrepresented among the DMR-associated genes in N22/IR64 and PK/IR64, respectively (Fig. 6b). The biological processes, regulation of gene expression, response to abiotic stimulus and chromatin binding were significant among the genes exhibiting on/off gene expression with respect to their methylation status (Supplementary Fig. S8c). Many of these genes represented known abiotic stress-responsive genes. For example, genes encoding for transcription factors (MYB, AP2-EREBP, WRKY, NAC and HB families), sodium transporter HKT1 homologs, F-box, calcium-dependent protein kinases, proteinases, peptidases, oxidoreductase, glutathione S-transferase, histone deacetylase and putative dicer-like proteins, were differentially methylated in N22 and/or Pokkali cultivars as compared to IR64 (Supplementary Table S4).

A few representative examples of differential methylation of genes involved in abiotic stress response and epigenetic regulation of gene expression have been shown in Fig. 6c. The transcript abundance of these proteins were found to be negatively correlated with their differential methylation status. For example, transcription factors, such as A20/AN1-like zinc finger (Os05g23470), homeobox (Os02g43330 and Os03g43930) and AGL19 (Os10g39130) exhibited differential methylation in N22/IR64 and/or PK/IR64. The methylation status of these transcription factors can regulate the expression of a cascade of several downstream targets. In addition, genes encoding calcium-dependent protein kinase, calmodulin, transporter protein, drought-responsive proteins, detoxifying enzymes (oxidoreductase, glutathione S-transferase and thioredoxin), F-box protein, and heat-shock proteins also exhibited differential methylation status and transcript abundance in the rice cultivars (Fig. 6c). The role of many of these genes in drought/salinity stress response has already been demonstrated. Interestingly, we observed differential methylation of many of the genes involved in epigenetic regulation of gene expression. For example, histone deacetylase (Os08g25570) was hypermethylated (downregulated), but histone methyltransferase (Os01g70220) was hypomethylated (upregulated) in N22. One of the RNA-directed DNA methylation protein (Os06g42430) was found to be hypermethylated with lower transcript abundance in Pokkali. Many of the genes encoding components of gene silencing machinery, such as dicer-like proteins (Os09g14610, Os08g05320 and Os04g43050) exhibited differential methylation and transcript abundance in the rice cultivars. Although the differential expression of a few of these genes have been reported earlier^43^,^44^, their differential methylation was not known. These proteins are required for establishment and maintenance of different context cytosine methylation and many aspects of epigenetic regulation. Although the exact mechanism of involvement of these proteins remains to be elucidated, our results provide evidence for their role in environmental stress responses.

### Methylation status of TEs is correlated with transcription of proximal protein-coding genes

**Figure 7:**
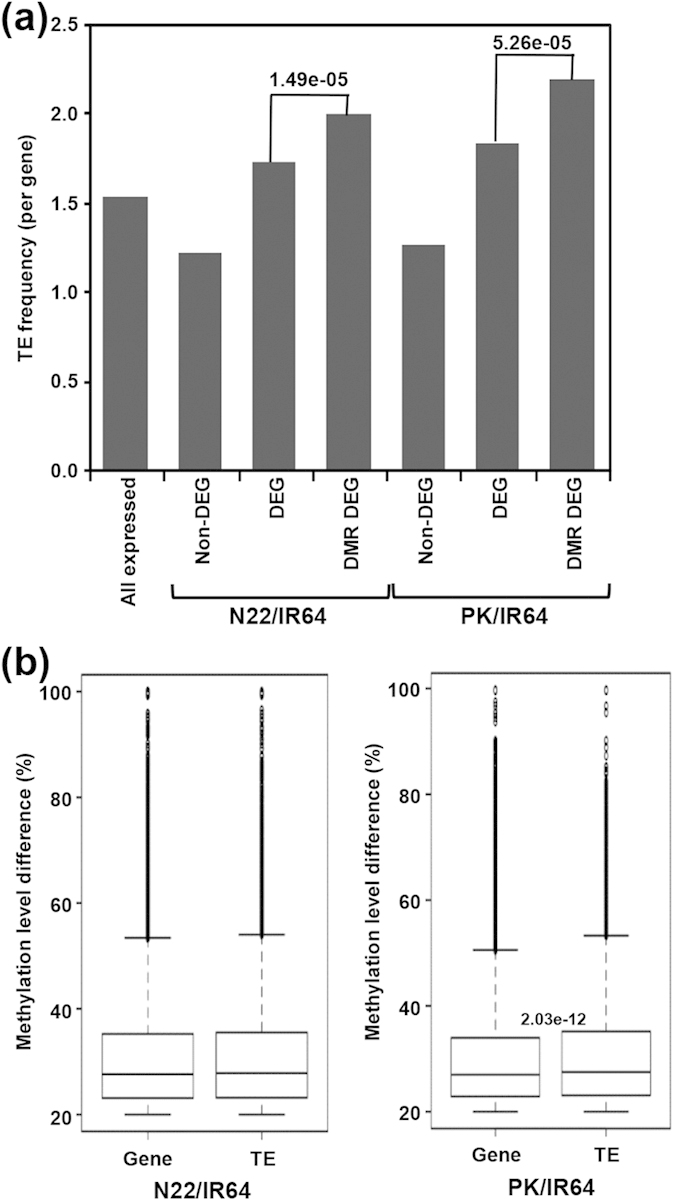
Correlation between differential expression and frequency/methylation level of TEs. (**a**) Average number of TEs per gene (within 2 Kb flanking sequences) for the indicated gene class. Non-DEG, non-differentially expressed genes; DEG, differentially expressed genes; DMR-DEG, differentially expressed genes associated with DMRs. (**b**) Box-plot showing distribution of methylation level difference of the differentially methylated regions (DMRs) associated with protein-coding genes and transposable elements (TEs) in different rice cultivars. *P*-value (Fisher’s exact text) of significant methylation difference is given. N22, Nagina 22; PK, Pokkali.

Further, we examined the possibility of involvement of methylation within TEs to regulate gene expression in different cultivars. We determined the relative TE density at DMR-associated DEGs or non-DEGs as compared to all DEGs or non-DEGs, respectively, for N22/IR64 and PK/IR64. The enrichment of TEs was substantially higher for DMR-associated DEGs as compared to non-DEGs for both N22/IR64 (*P*-value 1.49e-05) and PK/IR64 (*P*-value 5.26e-05) (Fig. 7a). However, TE density for all expressed genes and DEGs was lower. Further, we analyzed the methylation level difference of DMRs associated with protein-coding genes and TEs. We found significantly higher differences in the methylation level of DMRs associated with TEs as compared to protein-coding genes for PK/IR64 (Fig. 7b).

### Validation of DNA methylation and gene expression

To validate the results of differential methylation patterns, we selected a series of DMR candidate regions. Based on the whole-genome bisulphite-sequencing results, we picked at least 15 genomic regions (DMRs) lying within the genes known to be involved in abiotic stress response and epigenetic regulation of gene expression. In these regions, we performed traditional bisulphite-PCR followed by Sanger sequencing for the three rice cultivars. Notably, more than 90–95% of the methyl cytosines at CG sites were validated, whereas 80–90% and 75–87% of CHG and CHH sites, respectively, could be validated in the three rice cultivars. The correlation between methylation levels of all methyl cytosines ranged from 0.78–0.83 for CG, 0.73–0.77 for CHG and 0.70–0.71 for CHH context (Supplementary Fig. S9). In addition, about 70% of genes/loci reported to be differentially methylated in different stress-related rice cultivars in earlier studies^20^,^26^,^27^ were represented in our dataset.

Further, we validated transcript levels of at least 16 DMR-associated genes in rice cultivars by real-time PCR analysis. A high correlation (Pearson correlation 0.75) between the differential gene expression results from RNA-seq and qRT-PCR data, was obtained (Supplementary Fig. S10). Overall, the results were in concordance with results obtained from whole genome bisulphite sequencing and RNA-seq data analyses indicating high-quality of our data.

### Role of small RNAs in DNA methylation

**Figure 8:**
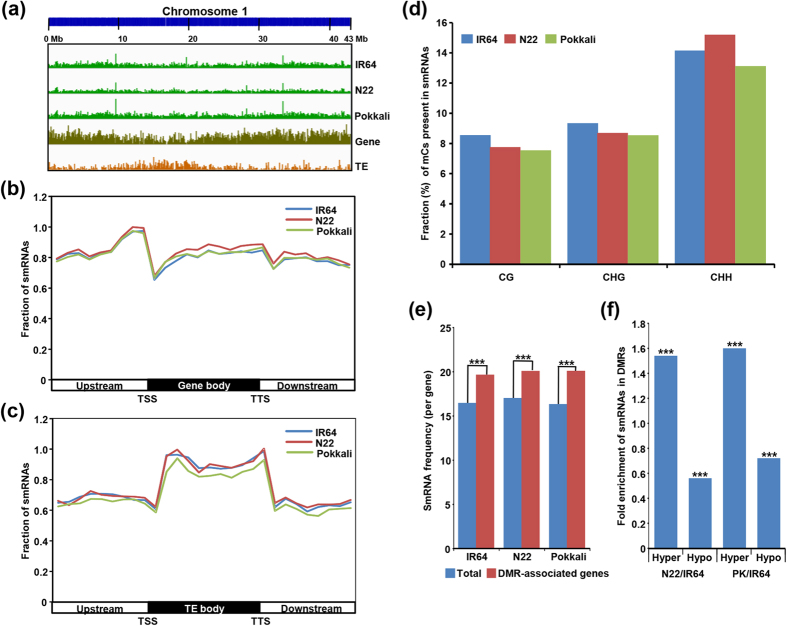
Genomic distribution of smRNAs and their relation with differential DNA methylation in rice cultivars. (**a**) The density of small RNAs, genes and transposable elements (TEs) throughout chromosome 1 (bin size 100 kb). (**b**,**c**) Relative fraction of smRNAs within gene (**b**) and TE (**c**) body and 1 kb flanking (upstream and downstream) sequences in each sequence context in different rice cultivars. TSS, transcriptional start site and TTS, transcriptional termination site. (**d**) Fraction of different contexts methylcytosines (mCs) present within the identified smRNAs. (**e**) Frequency of smRNAs in total genes and DMR-associated genes. (**f**) Enrichment of smRNAs positioned within hyper and hypo DMRs. Significance (Fisher’s exact text) of enrichment has been marked with asterisks ***P < 1 × 10^−20^). N22, Nagina 22; PK, Pokkali.

A correlation between small RNA (smRNA) abundance and DNA methylation has been established by many studies^10^,^18^,^29^,^32^. Therefore, we sought to investigate the relationship between DNA methylation and smRNAs in the rice cultivars. To perform genome-wide discovery of smRNAs, small RNA libraries for IR64, N22 and Pokkali seedlings were sequenced and more than 15 million reads were generated for each sample. After pre-processing, the unique reads (smRNAs) of 21–24 nt were aligned to the rice genome (Supplementary Table S5). A total of 46–47% of the smRNAs (21–24 nt) mapped uniquely to the rice genome. The small RNAs were well distributed throughout the rice genome without any obvious difference in the different cultivars (Fig. 8a, Supplementary Fig. S2). The integration of genomic coordinates of smRNAs with the rice genome annotation revealed that a large fraction (68–70%) of smRNAs were originated from the genic and flanking sequences. The abundance of smRNAs was higher in the upstream sequences as compared to the gene body and downstream sequences (Fig. 8b). Highest abundance of smRNAs was observed in the upstream sequences near the TSSs. Within the genebody, highest abundance of smRNAs was observed at the 3′-end. However, the abundance of smRNAs was much higher within TE body as compared to the upstream and downstream sequences (Fig. 8c). This pattern seems to be consistent with the CHH methylation patterns in protein-coding genes and TEs (Fig. 2b,c). Further, we searched for the presence of mCs in different contexts within the smRNA loci. Overall 9–10% of total mCs were associated with smRNA loci in different rice cultivars and 53–55% of smRNA loci contained at least one mC. Notably, the proportion of CHH context mCs lying within smRNAs (13.1–15.2%) was substantially higher as compared to CG (7.5–8.5%) and CHG (8.5–9.3%) context mCs in all the three rice cultivars (Fig. 8d). The higher frequency of CHH context mCs located within the smRNA loci suggested a positive correlation between them.

Next, we investigated the correlation between smRNA abundance and differential DNA methylation. We estimated the abundance of smRNA in all the protein-coding genes and DMR-associated genes in the rice cultivars. We found that abundance of smRNAs was significantly higher in the DMR-associated genes in all the three cultivars (Fig. 8e). In addition, we noticed significantly higher (~1.6–1.8 fold) enrichment of smRNAs in the hypermethylated genes and significant (~two-fold) depletion of smRNAs in the hypomethylated genes in both N22/IR64 and PK/IR64 comparisons (Fig. 8f). These results suggest that smRNAs participate in alteration of DNA methylation levels in different rice cultivars.

## Discussion

Growing evidences from recent studies have suggested that DNA methylation plays a crucial role in regulation of stress responses/adaptation in plants^15^,^26^,^45^,^46^. Rice has rich germplasm resources with variability in their adaptive response to environmental cues, such as drought and salinity. The epigenetic marks, such as DNA methylation may be responsible for phenotypic consequences like tolerance to abiotic stresses. Therefore, it is important to investigate the DNA methylomes of rice cultivars with contrasting phenotype towards abiotic stress tolerance to understand the epigenetic regulation of stress adaptation and identify specific marks that contribute to the modulation of this agronomic trait.

In this study, we assessed the dynamics of DNA methylation at single base resolution in rice cultivars with contrasting drought and salinity stress response. Our results revealed several features regarding distribution of mCs, differential methylation patterns and their relationship with gene expression. The highest fraction of mCs in CG context followed by CHG and CHH contexts observed in the rice cultivars seems to be due to higher statistical power in detecting change at CG sites followed by CHG and CHH sites, which follow the same pattern of average methylation levels^28^,^32^. Significant differences were observed in the methylation patterns in different rice cultivars with contrasting stress response. We found several cultivar-specific methylated regions in the rice genome and detected genome-wide differences in DNA methylation status, which were coupled to the gene expression, strengthening the possible role of epigenetic mechanisms in abiotic stress adaptation. Significant differences in methylation patterns of specific loci have been reported in different cultivars/lines with contrasting phenotype under stress conditions^26^,^27^,^46^,^47^. Hypomethylation/demethylation is considered as a common feature associated with adaptive response to various stresses^19^,^47^,^48^,^49^. Based on methylation sensitive amplified polymorphism analysis, it has been shown that hypomethylation and hypermethylation are more frequent in drought-tolerant and drought-sensitive rice genotypes, respectively, under drought stress conditions^50^. Other studies also suggested the genotype and developmental/tissue specificity of epigenetic regulation of abiotic stress responses in rice cultivars^26^,^27^. Differential methylation in IR64, N22 and Pokkali rice cultivars under stress conditions is also expected to contribute to their contrasting stress response phenotype. Overall, these results suggest cultivar-specific changes in DNA methylation and different unknown mechanism(s) might be responsible for the differential stress responses.

Our genome-wide analysis provide unique insight into the dynamics of DNA methylation in rice cultivars. The results suggest that DNA methylation regulate stress responsive genes via altering their expression. Our observations are consistent with previous reports associating methylation status of the DNA with transcriptional control of specific loci in different plant species^18^,^48^,^51^,^52^,^53^. Although we found significant enrichment of down and upregulated genes associated with hyper and hypo-DMRs, respectively, a large fraction of the DEGs did not exhibit significant differences in their methylation levels among cultivars. It may be speculated that the effect of DNA methylation on gene expression may be mediated either directly or indirectly via some transcriptional regulatory proteins, which can recognize mCs in the promoter regions. A mechanism involving the recruitment of methyl CG-binding proteins to remodel chromatin by utilizing histone deacetylase activity has been proposed that regulate gene expression^54^,^55^,^56^,^57^. An alternate mechanism of gene silencing via inhibition of transcription activator binding due to promoter DNA methylation has also been reported earlier^58^,^59^,^60^. Further, we observed a stronger correlation of gene body methylation/demethylation as compared to that of flanking sequences with decreased/increased transcript abundance. These results are consistent with previous reports, which demonstrated that DNA methylation in gene bodies affects gene expression more effectively than the promoter methylation in various organisms including rice^18^,^48^,^61^,^62^,^63^. However, these observations are in contrast with other studies, which reported a strong correlation between promoter methylation and gene expression^42^,^64^. Further studies are required to clarify that how the transcript abundance is regulated differentially by the gene body or promoter methylation in different biological contexts. Overall, the identification of diverse categories of genes including those involved in abiotic stress response with differential methylation patterns provides evidence that epigenetic modifications play a crucial role in plants adaptation/response to abiotic stresses.

The association of DNA methylation with inactivation of transposon activity is well known^65^,^66^. Several genome-wide studies have shown heavy methylation of transposon sequences^67^,^68^. It is now well established that the position and methylation status of nearby transposon(s) can regulate the gene expression^39^,^42^,^69^,^70^,^71^. We also observed heavy methylation of TEs in rice cultivars and their methylation patterns were quite different than protein-coding genes. Higher methylation of TEs is due to their ability to recruit the silencing machinery and is considered as an evolutionary mechanism to silence their expression and mobility^65^,^66^. Many of the DMRs were associated with TEs and changes in the methylation of TEs was coupled to the expression of proximal protein-coding genes. Similar results have been reported in other studies on different plants as well^18^,^42^,^72^. A few evidences have demonstrated that abiotic stresses can evoke heritable changes in the epigenetic framework that can confer enhanced stress tolerance in the progeny due to transgenerational memory^45^,^73^,^74^. Interestingly, a copia-type retrotransposon (*ONSEN*) in Arabidopsis was found to have heat-induced transcription and transposition activity, which was found to be transgenerationally inherited^75^. Altogether, it can be speculated that changes in DNA methylation patterns can remove epigenetic constraints from TEs, thereby resulting in transcriptional changes in stress-responsive genes.

The *de novo* DNA methylation is established mainly via small RNA (smRNA)-guided RdDM pathway^76^,^77^. Recently, bisulphite sequencing of selected genes in *rdd* mutant (a triple DNA demethylase mutant) Arabidopsis plants revealed that RdDM-associated CHH methylation play a positive role in stress-responsive gene expression^42^. We observed a concordance in distribution patterns of mCs in CHH context and smRNAs within gene/TE body and their flanking sequences in the rice cultivars. In addition, a larger fraction of CHH context mCs were associated with smRNA loci. As CHH methylation is established by *de novo* methyltransferases only, our data also suggested a larger participation of smRNAs in CHH methylation which can regulate the target gene expression. Further, smRNAs were significantly enriched in hypermethylated regions, but depleted in hypomethylated regions. Overall, these results suggest that smRNAs also play a crucial role in shaping the DNA methylation landscape.

## Conclusions

Our results suggest the potential role of cultivar-specific DNA methylation patterns as an important regulatory mechanism for sensing and responding to the stress conditions via modulation of stress-responsive gene expression. Our investigation suggest that variability for drought/salinity tolerance in rice germplasm is dependent on the extent and patterns of DNA methylation. We have provided evidence suggesting that DNA methylation play an important role in abiotic stress adaptation/response by regulating expression of a set of stress-responsive genes in rice largely via methylation/demethylation of proximal TEs. The DMRs described here provide an initial set of targets of epigenetic variations across rice germplasm and may provide basis of future selection strategies. Further, it would be interesting to study the changes in methylation pattern in the rice cultivars under stress conditions. Overall, the understanding of epigenetic regulation of abiotic stress responses can have a significant impact in breeding for development of improved varieties with enhanced stress tolerance.

## Methods

### Plant material and genomic DNA isolation

Three rice (*Oryza sativa*) cultivars, IR64 (drought and salinity sensitive), Pokkali (salinity-tolerant) and Nagina 22 (drought-tolerant), were used for DNA methylome analysis in this study. Rice seeds after surface sterilization with sodium hypochlorite solution were grown in hydroponics in a culture room under 14 h light/10 h dark conditions at 28 ± 1 °C. After two weeks of growth, seedlings of different cultivars were harvested, frozen in liquid nitrogen and stored at −80 °C till further use. The experimental set-up was repeated to collect three independent biological replicates of each tissue sample. Genomic DNA was isolated from frozen tissues using Qiagen DNeasy Minikit (Qiagen) as per manufacturer’s protocol. The quality and quantity of the genomic DNA samples were checked by Nanodrop Spectrophotometer (Thermo Scientific) and Qubit Fluorimeter (Life Technologies). Agarose gel electrophoresis was also carried out to ascertain the quality of genomic DNA samples.

### Whole genome bisulphite sequencing

The genomic DNA isolated from IR64, N22 and Pokkali seedlings (pooled in equal quantity from the three independent biological replicates) were processed for bisulphite sequencing. The genomic DNA samples were fragmented via sonication to a size of 100–300 bp, end repaired and TruSeq-methylated adapters were ligated to the DNA fragments. Approximately, 500 ng of adapter-ligated DNA fragments were used for bisulphite conversion using EZ DNA Methylation-Gold^TM^ kit (Zymo Research Corporation, CA, USA) according to manufacturer’s protocol. After desalting, size selection and PCR amplification, library quality was analyzed. The qualified libraries were sequenced on the HiSeq 2000 system (Illumina) for 90 cycles in paired-end mode to achieve more than 30 × genome coverage for each sample and processed sequenced data (after removal of reads containing adaptor sequences and low-quality reads) was obtained. The sequence data were further filtered using our in-house NGS QC Toolkit (v2.3)^78^.

### Read alignment and identification of mCs

The filtered 90 bp paired-end reads from each sample were aligned to the rice genome sequence (MSU v7.0) using Bismark (v0.8)^79^ under default parameters. Reads from each sample were processed to remove clonal reads by retaining only one of such reads. Only the reads aligned at unique location in the genome were retained. Alignment of all the reads was performed on the rice chloroplast genome also to estimate the bisulphite conversion efficiency and error-rate. A bisulphite conversion efficiency of ≥99% was observed for all the samples. The non-conversion rate of chloroplast genome Cs was considered as a measure of false discovery rate (error rate). The alignment files on the rice genome were used as input in Methylkit (v0.5.6) for further analysis. The mCs were identified by *P*-value calculation using binomial distribution [P = binomial (m, x, error_rate), where m = number of reads giving methylated call, n = number of reads giving unmethylated call; x = m + n, the sequencing depth of single C and error_rate was the error rate for bisulphite nonconversion of the sample] as described earlier^37^. This step excludes mCs, which might be the result of non-conversion of cytosines during bisulphite conversion. A *P*-value cut-off of 0.001 and minimum read-depth of five was used to identity true mCs. The methylation level (percentage of reads showing mC among all the reads covering the same cytosine site) of each identified mC was determined. Various analyses, including coverage determination, distribution of mCs in the genome and various genomic features, and determination of frequency of flanking nucleotides were carried out using custom perl scripts.

### Identification of DMRs

For screening of DMRs, we determined the frequency of mCs in each bin size of 100 bp throughout the genome. Only the cytosine sites covered by at least five reads in each sample were considered. For each bin, the methylation level at each cytosine site was calculated for each sample. The bins containing at least three mCs and a minimum difference of 20% methylation level with *q*-value of 0.01 were identified as DMRs. For estimation of *q*-value, *P*-value was determined by Fisher’s exact test followed by correction using Sliding Linear Model (SLIM). The DMRs were positioned within gene body or 2 kb flanking sequences based on the position of mid-point relative to gene position coordinates.

### Gene ontology (GO) enrichment analysis

The enrichment analysis of GO categories within the methylated genes (methylation at promoters and/or within annotated transcribed regions) was performed using the BiNGO tool and visualized using Cytoscape (v3.7). The GO categories with *P*-value 0.05 after applying FWER correction were considered as significantly enriched.

### RNA sequencing and data analysis

Total RNA was extracted using TRI Reagent (Sigma Life Science, USA), according to manufacturer’s instructions. The quality and quantity of RNA samples were assessed using Agilent Bioanalyzer (Agilent Technologies, Singapore) as described previously^80^. For sequencing, cDNA libraries were generated from total RNA of each tissue sample and sequencing was performed on Illumina platform to generate 100 bp paired-end reads. The Fastq files were obtained and various quality controls were performed using NGS QC Toolkit^78^. Filtered high-quality reads were mapped on the rice genome (MSU7) using Tophat (v2.0.0) software. To analyze gene expression, a consensus reference-guided assembly of the transcriptome data from all samples was generated using Cufflinks (v2.0.2) and differential expression of genes among rice cultivars was determined by Cuffdiff as described^81^. Only the genes exhibiting significant difference (at least two-fold change with *P*-value ≤ 0.05) were considered.

### Methylation-expression correlation analysis

The correlation between methylation and gene expression was determined by comparison of methylation status of DMR-associated genes and their expression level/differential expression measured by RNA-seq. A box-and-whisker plot (boxplot R function) of the differential expression levels of the genes associated with hypo- or hyper-methylated DMRs and all rice genes was generated. The significance of differences was estimated using a two-tailed Wilcoxon rank-sum test (wilcox.test R function) using R programming environment.

### Small RNA sequencing and data analysis

The total RNAs isolated from seedlings of IR64, N22 and Pokkali rice cultivars were used for library preparation using TruSeq Small RNA Sample Prep Kit (Illumina Technologies) according to the manufacturer’s instructions. Each small RNA library was sequenced for 50 cycles on Illumina platform and the sequence data was obtained in FASTQ files for further processing. The sequence data was pre-processed using modified perl script as described previously^82^ and only the unique reads were retained. Further, all the unique reads from each sample were mapped to the rice genome sequence using Bowtie2. Only the uniquely mapped reads of 21–24 nt in length were used for subsequent analysis.

### Locus-specific bisulphite sequencing of selected DMRs

About 1 μg of genomic DNA extracted from unstressed control seedlings of rice genotypes was bisulphite converted using EZ DNA Methylation-Gold^TM^ kit (Qiagen). The bisulphite converted DNA was desalted and an aliquot of it was used for PCR amplification of the selected genomic regions representing DMRs using locus-specific forward and reverse primers. The purified PCR products were cloned in pGEM-T Easy vector (Promega) and confirmed clones were sequenced via Sanger sequencing method. An average of 10 clones were chosen randomly for sequencing. Methylation status of cytosines was assessed by comparing the sequence of the bisulphite treated DNA with that of untreated DNA.

### Quantitative PCR analysis

The gene-specific primers for qRT-PCR were designed using Primer Express (v3.0) software (Applied Biosystems, Foster City, CA). The primer sequences used in this study are listed in Supplementary Table S6. The quantitative PCR analysis was performed as described previously^80^ employing ABI 7500 Real-Time PCR System (Applied Biosystems). At least two independent biological replicates for each sample and three technical replicates of each biological replicate were analyzed for the analysis. The transcript level of each gene in different tissue samples was normalized with the transcript level of internal control gene, *Ubiquitin5* (*UBQ5*)^83^ and fold change was calculated as compared to the control condition.

### Data availability

The DNA methylation, RNA-seq and small RNA sequencing data generated in this study have been deposited with NCBI at Gene expression Omnibus (GEO) under series accession numbers GSE60288, GSE60287 and GSE64651, respectively.

## Additional Information

**How to cite this article**: Garg, R. *et al.* Divergent DNA methylation patterns associated with gene expression in rice cultivars with contrasting drought and salinity stress response. *Sci. Rep.* **5**, 14922; doi: 10.1038/srep14922 (2015).

## Supplementary Material

Supplementary Information

Supplementary Table S3

Supplementary Table S4

## References

[b1] Soppe W. J. *et al.* The late flowering phenotype of *fwa* mutants is caused by gain-of-function epigenetic alleles of a homeodomain gene. Mol. Cell 6, 791–802 (2000). 11090618 10.1016/s1097-2765(05)00090-0

[b2] Manning K. *et al.* A naturally occurring epigenetic mutation in a gene encoding an SBP-box transcription factor inhibits tomato fruit ripening. Nat. Genet. 38, 948–952 (2006). 16832354 10.1038/ng1841

[b3] Miura K. *et al.* A metastable *DWARF1* epigenetic mutant affecting plant stature in rice. Proc. Natl. Acad. Sci. USA 106, 11218–11223 (2009). 19541604 10.1073/pnas.0901942106 PMC2708680

[b4] Lindroth A. M. *et al.* Requirement of CHROMOMETHYLASE3 for maintenance of CpXpG methylation. Science 292, 2077–2080 (2001). 11349138 10.1126/science.1059745

[b5] Cao X. & Jacobsen S. E. Role of the Arabidopsis DRM methyltransferases in *de novo* DNA methylation and gene silencing. Curr. Biol. 12, 1138–1144 (2002). 12121623 10.1016/s0960-9822(02)00925-9

[b6] Kato M., Miura A., Bender J., Jacobsen S. E. & Kakutani T. Role of CG and non-CG methylation in immobilization of transposons in Arabidopsis. Curr. Biol. 13, 421–426 (2003). 12620192 10.1016/s0960-9822(03)00106-4

[b7] Hsieh T. F. *et al.* Genome-wide demethylation of Arabidopsis endosperm. Science 324, 1451–1454 (2009). 19520962 10.1126/science.1172417 PMC4044190

[b8] Gazzani S., Gendall A. R., Lister C. & Dean C. Analysis of the molecular basis of flowering time variation in Arabidopsis accessions. Plant Physiol. 132, 1107–1114 (2003). 12805638 10.1104/pp.103.021212 PMC167048

[b9] Liu J., He Y., Amasino R. & Chen X. siRNAs targeting an intronic transposon in the regulation of natural flowering behavior in Arabidopsis. Genes Dev. 18, 2873–2878 (2004). 15545622 10.1101/gad.1217304 PMC534648

[b10] Song Q. X. *et al.* Genome-wide analysis of DNA methylation in soybean. Mol. Plant 6, 1961–1974 (2013). 23966636 10.1093/mp/sst123

[b11] Saze H., MittelstenScheid O. & Paszkowski J. Maintenance of CpG methylation is essential for epigenetic inheritance during plant gametogenesis. Nat. Genet. 34, 65–69 (2003). 12669067 10.1038/ng1138

[b12] Gehring M., Bubb K. L. & Henikoff S. Extensive demethylation of repetitive elements during seed development underlies gene imprinting. Science 324, 1447–1451 (2009). 19520961 10.1126/science.1171609 PMC2886585

[b13] Zemach A. *et al.* Local DNA hypomethylation activates genes in rice endosperm. Proc. Natl. Acad. Sci. USA 107, 18729–18734 (2010). 20937895 10.1073/pnas.1009695107 PMC2972920

[b14] Chen X. & Zhou D. X. Rice epigenomics and epigenetics: challenges and opportunities. Curr. Opin. Plant Biol. 16, 164–169 (2013). 23562565 10.1016/j.pbi.2013.03.004

[b15] Chinnusamy V. & Zhu J. K. Epigenetic regulation of stress responses in plants. Curr. Opin. Plant Biol. 12, 133–139 (2009). 19179104 10.1016/j.pbi.2008.12.006 PMC3139470

[b16] Mirouze M. *et al.* Selective epigenetic control of retrotransposition in Arabidopsis. Nature 461, 427–430 (2009). 19734882 10.1038/nature08328

[b17] Lang-Mladek C. *et al.* Transgenerational inheritance and resetting of stress-induced loss of epigenetic gene silencing in Arabidopsis. Mol. Plant 3, 594–602 (2010). 20410255 10.1093/mp/ssq014 PMC2877484

[b18] Dowen R. H. *et al.* Widespread dynamic DNA methylation in response to biotic stress. Proc. Natl. Acad. Sci. USA 109, E2183–2191 (2012). 22733782 10.1073/pnas.1209329109 PMC3420206

[b19] Uthup T. K., Ravindran M., Bini K. & Thakurdas S. Divergent DNA methylation patterns associated with abiotic stress in *Heveabrasiliensis*. Mol. Plant 4, 996–1013 (2011). 21705581 10.1093/mp/ssr039

[b20] Shaik R. & Ramakrishna W. Bioinformatic analysis of epigenetic and microRNA mediated regulation of drought responsive genes in rice. PLoS ONE 7, e49331 (2012). 23145152 10.1371/journal.pone.0049331 PMC3493535

[b21] Bilichak A., Ilnystkyy Y., Hollunder J. & Kovalchuk I. The progeny of *Arabidopsis thaliana* plants exposed to salt exhibit changes in DNA methylation, histone modifications and gene expression. PLoS ONE 7, e30515 (2012). 22291972 10.1371/journal.pone.0030515 PMC3264603

[b22] McNally K. L. *et al.* Genomewide SNP variation reveals relationships among landraces and modern varieties of rice. Proc. Natl. Acad. Sci. USA 106, 12273–12278 (2009). 19597147 10.1073/pnas.0900992106 PMC2718348

[b23] Nakashima K., Ito Y. & Yamaguchi-Shinozaki K. Transcriptional regulatory networks in response to abiotic stresses in Arabidopsis and grasses. Plant Physiol. 149, 88–95 (2009). 19126699 10.1104/pp.108.129791 PMC2613698

[b24] Bhattacharjee A. & Jain M. Transcription factor mediated abiotic stress signaling in rice. In: Plant Stress - Stress-Mediated Signaling in Plants (Pandey G. K. ed.) 7, 16–25. Global Science Books, Academic Press, Japan (2013).

[b25] Jain M., Moharana K. C., Shankar R., Kumari R. & Garg R. Genome wide discovery of DNA polymorphisms in rice cultivars with contrasting drought and salinity stress response and their functional relevance. Plant Biotechnol. J. 12, 253–264 (2014). 24460890 10.1111/pbi.12133

[b26] Wang W. S. *et al.* Drought-induced site-specific DNA methylation and its association with drought tolerance in rice (*Oryza sativa* L.). J. Exp. Bot. 62, 1951–1960 (2011). 21193578 10.1093/jxb/erq391 PMC3060682

[b27] Karan R., DeLeon T., Biradar H. & Subudhi P. K. Salt stress induced variation in DNA methylation pattern and its influence on gene expression in contrasting rice genotypes. PLoS ONE 7, e40203 (2012). 22761959 10.1371/journal.pone.0040203 PMC3386172

[b28] Cokus S. J. *et al.* Shotgun bisulphite sequencing of the Arabidopsis genome reveals DNA methylation patterning. Nature 452, 215–219 (2008). 18278030 10.1038/nature06745 PMC2377394

[b29] Calarco J. P. *et al.* Reprogramming of DNA methylation in pollen guides epigenetic inheritance via small RNA. Cell 151, 194–205 (2012). 23000270 10.1016/j.cell.2012.09.001 PMC3697483

[b30] Zhong S. *et al.* Single-base resolution methylomes of tomato fruit development reveal epigenome modifications associated with ripening. Nat. Biotechnol. 31, 154–159 (2013). 23354102 10.1038/nbt.2462

[b31] Beck S. & Rakyan V. K. The methylome: approaches for global DNA methylation profiling. Trends Genet. 24, 231–237 (2008). 18325624 10.1016/j.tig.2008.01.006

[b32] Lister R. *et al.* Highly integrated single-base resolution maps of the epigenome in Arabidopsis. Cell 133, 523–536 (2008). 18423832 10.1016/j.cell.2008.03.029 PMC2723732

[b33] Schmitz R. J. & Zhang X. High-throughput approaches for plant epigenomic studies. Curr. Opin. Plant Biol. 14, 130–136 (2011). 21470901 10.1016/j.pbi.2011.03.010 PMC3112054

[b34] Kim K. D., El, Baidouri M. & Jackson S. A. Accessing epigenetic variation in the plant methylome. *Brief. Funct*. Genomics 13, 318–327 (2014). 10.1093/bfgp/elu003 24562692

[b35] Walia H. *et al.* Comparative transcriptional profiling of two contrasting rice genotypes under salinity stress during the vegetative growth stage. Plant Physiol. 139, 822–835 (2005). 16183841 10.1104/pp.105.065961 PMC1255998

[b36] Lenka S. K., Katiyar A., Chinnusamy V. & Bansal K. C. Comparative analysis of drought-responsive transcriptome in Indica rice genotypes with contrasting drought tolerance. Plant Biotechnol. J. 9, 315–327 (2011). 20809928 10.1111/j.1467-7652.2010.00560.x

[b37] Greaves I. K. *et al.* Trans chromosomal methylation in Arabidopsis hybrids. Proc. Natl. Acad. Sci. USA 109, 3570–3575 (2012). 22331882 10.1073/pnas.1201043109 PMC3295253

[b38] Law J. A. & Jacobsen S. E. Establishing, maintaining and modifying DNA methylation patterns in plants and animals. Nat. Rev. Genet. 11, 204–220 (2010). 20142834 10.1038/nrg2719 PMC3034103

[b39] Gent J. I. *et al.* CHH islands: *de novo* DNA methylation in near-gene chromatin regulation in maize. Genome Res. 23, 628–637 (2013). 23269663 10.1101/gr.146985.112 PMC3613580

[b40] Kawasaki S. *et al.* Gene expression profiles during the initial phase of salt stress in rice. Plant Cell 13, 889–905 (2001). 11283343 10.1105/tpc.13.4.889 PMC135538

[b41] Ray S. *et al.* Modulation of transcription factor and metabolic pathway genes in response to water-deficit stress in rice. Funct. Integr. Genomics 11, 157–178 (2011). 20821243 10.1007/s10142-010-0187-y

[b42] Le T. N. *et al.* DNA demethylases target promoter transposable elements to positively regulate stress responsive genes in Arabidopsis. Genome Biol. 15, 458 (2014). 25228471 10.1186/s13059-014-0458-3 PMC4189188

[b43] Kapoor M. *et al.* Genome-wide identification, organization and phylogenetic analysis of Dicer-like, Argonaute and RNA-dependent RNA Polymerase gene families and their expression analysis during reproductive development and stress in rice. BMC Genomics 9, 451 (2008). 18826656 10.1186/1471-2164-9-451 PMC2576257

[b44] Sharma R. *et al.* Rice cytosine DNA methyltransferases-gene expression profiling during reproductive development and abiotic stress. FEBS J. 276, 6301–6311 (2009). 19788421 10.1111/j.1742-4658.2009.07338.x

[b45] Boyko A. *et al.* Transgenerational adaptation of Arabidopsis to stress requires DNA methylation and the function of Dicer-like proteins. PLoS ONE 5, e9514 (2010). 20209086 10.1371/journal.pone.0009514 PMC2831073

[b46] Zheng X. *et al.* Transgenerational variations in DNA methylation induced by drought stress in two rice varieties with distinguished difference to drought resistance. PLoS ONE 8, e80253 (2013). 24244664 10.1371/journal.pone.0080253 PMC3823650

[b47] Wang M. *et al.* Induced and constitutive DNA methylation in a salinity-tolerant wheat introgression line. Plant Cell Physiol. 55, 1354–1365 (2014). 24793752 10.1093/pcp/pcu059

[b48] Choi C. S. & Sano H. Abiotic-stress induces demethylation and transcriptional activation of a gene encoding a glycerophosphodiesterase-like protein in tobacco plants. Mol. Genet. Genomics 277, 589–600 (2007). 17273870 10.1007/s00438-007-0209-1

[b49] Angers B., Castonguay E. & Massicotte R. Environmentally induced phenotypes and DNA methylation: how to deal with unpredictable conditions until the next generation and after. Mol. Ecol. 19, 1283–1295 (2010). 20298470 10.1111/j.1365-294X.2010.04580.x

[b50] Gayacharan & Joel A. J. Epigenetic responses to drought stress in rice (*Oryza sativa* L.). Physiol. Mol. Biol. Plants 19, 379–387 (2013). 24431506 10.1007/s12298-013-0176-4 PMC3715639

[b51] Steward N., Ito M., Yamaguchi Y., Koizumi N. & Sano H. Periodic DNA methylation in maize nucleosomes and demethylation by environmental stress. J. Biol. Chem. 277, 37741–37746 (2002). 12124387 10.1074/jbc.M204050200

[b52] Wada Y., Miyamoto K., Kusano T. & Sano H. Association between up-regulation of stress-responsive genes and hypomethylation of genomic DNA in tobacco plants. Mol. Genet. Genomics 271, 658–666 (2004). 15148604 10.1007/s00438-004-1018-4

[b53] Zilberman D., Gehring M., Tran R. K., Ballinger T. & Henikoff S. Genome-wide analysis of *Arabidopsis thaliana* DNA methylation uncovers an interdependence between methylation and transcription. Nat. Genet. 39, 61–69 (2007). 17128275 10.1038/ng1929

[b54] Jones P. L. *et al.* Methylated DNA and MeCP2 recruit histone deacetylase to repress transcription. Nat. Genet. 19, 187–191 (1998). 9620779 10.1038/561

[b55] Nan X. *et al.* Transcriptional repression by the methyl-CpG-binding protein MeCP2 involves a histone deacetylase complex. Nature 393, 386–389 (1998). 9620804 10.1038/30764

[b56] Ng H. H. & Bird A. DNA methylation and chromatin modification. Curr. Opin. Genet. Dev. 9, 158–163 (1999). 10322130 10.1016/s0959-437x(99)80024-0

[b57] Clouaire T. & Stancheva I. Methyl-CpG binding proteins: specialized transcriptional repressors or structural components of chromatin? Cell Mol. Life Sci. 65, 1509–1522 (2008). 18322651 10.1007/s00018-008-7324-y PMC2873564

[b58] Watt F. & Molloy P. L. Cytosine methylation prevents binding to DNA of a HeLa cell transcription factor required for optimal expression of the adenovirus major late promoter. Genes Dev. 2, 1136–1143 (1988). 3192075 10.1101/gad.2.9.1136

[b59] Bird A. DNA methylation patterns and epigenetic memory. Genes Dev. 16, 6–21 (2002). 11782440 10.1101/gad.947102

[b60] Scebba F. *et al.* Arabidopsis MBD proteins show different binding specificities and nuclear localization. Plant Mol. Biol. 53, 715–731 (2003). 15010609 10.1023/B:PLAN.0000019118.56822.a9

[b61] Hohn T., Corsten S., Rieke S., Müller M. & Rothnie H. Methylation of coding region alone inhibits gene expression in plant protoplasts. Proc. Natl. Acad. Sci. USA 93, 8334–8339 (1996). 8710871 10.1073/pnas.93.16.8334 PMC38671

[b62] Wu H. *et al.* Dnmt3a-dependent nonpromoter DNA methylation facilitates transcription of neurogenic genes. Science 329, 444–448 (2010). 20651149 10.1126/science.1190485 PMC3539760

[b63] Wang Y., Wang X., Lee T. H., Mansoor S. & Paterson A. H. Gene body methylation shows distinct patterns associated with different gene origins and duplication modes and has a heterogeneous relationship with gene expression in *Oryza sativa* (rice). New Phytol. 198, 274–283 (2013). 23356482 10.1111/nph.12137

[b64] Yamamuro C. *et al.* Overproduction of stomatal lineage cells in Arabidopsis mutants defective in active DNA demethylation. Nat. Commun. 5, 4062 (2014). 24898766 10.1038/ncomms5062 PMC4097119

[b65] Slotkin R. K. & Martienssen R. Transposable elements and the epigenetic regulation of the genome. Nat. Rev. Genet. 8, 272–285 (2007). 17363976 10.1038/nrg2072

[b66] Lisch D. Epigenetic regulation of transposable elements in plants. Annu. Rev. Plant Biol. 60, 43–66 (2009). 19007329 10.1146/annurev.arplant.59.032607.092744

[b67] Liu S. *et al.* Mu transposon insertion sites and meiotic recombination events co-localize with epigenetic marks for open chromatin across the maize genome. PLoS Genet. 5, e1000733 (2009). 19936291 10.1371/journal.pgen.1000733 PMC2774946

[b68] Zemach A., McDaniel I. E., Silva P. & Zilberman D. Genome-wide evolutionary analysis of eukaryotic DNA methylation. Science 328, 916–919 (2010). 20395474 10.1126/science.1186366

[b69] Lippman Z. *et al.* Role of transposable elements in heterochromatin and epigenetic control. Nature 430, 471–476 (2004). 15269773 10.1038/nature02651

[b70] Hollister J. D. & Gaut B. S. Epigenetic silencing of transposable elements: a trade-off between reduced transposition and deleterious effects on neighboring gene expression. Genome Res. 19, 1419–1428 (2009). 19478138 10.1101/gr.091678.109 PMC2720190

[b71] Ahmed I., Sarazin A., Bowler C., Colot V. & Quesneville H. Genome-wide evidence for local DNA methylation spreading from small RNA-targeted sequences in Arabidopsis. Nucleic Acids Res. 39, 6919–6931 (2011). 21586580 10.1093/nar/gkr324 PMC3167636

[b72] Stroud H. *et al.* Plants regenerated from tissue culture contain stable epigenome changes in rice. Elife 2, e00354 (2013). 23539454 10.7554/eLife.00354 PMC3601819

[b73] Ou X. *et al.* Transgenerational inheritance of modified DNA methylation patterns and enhanced tolerance induced by heavy metal stress in rice (*Oryza sativa* L.). PLoS ONE 7, e41143 (2012). 22984395 10.1371/journal.pone.0041143 PMC3439459

[b74] Pecinka A. & MittelstenScheid O. Stress-induced chromatin changes: a critical view on their heritability. Plant Cell Physiol. 53, 801–808 (2012). 22457398 10.1093/pcp/pcs044 PMC3345370

[b75] Ito H. *et al.* An siRNA pathway prevents transgenerational retrotransposition in plants subjected to stress. Nature 472, 115–119 (2011). 21399627 10.1038/nature09861

[b76] Cao X. & Jacobsen S. E. Locus-specific control of asymmetric and CpNpG methylation by the DRM and CMT3 methyltransferase genes. Proc. Natl. Acad. Sci. USA 99, 16491–16498 (2002). 12151602 10.1073/pnas.162371599 PMC139913

[b77] Zheng X., Zhu J., Kapoor A. & Zhu J. K. Role of Arabidopsis AGO6 in siRNA accumulation, DNA methylation and transcriptional gene silencing. EMBO J. 26, 1691–1701 (2007). 17332757 10.1038/sj.emboj.7601603 PMC1829372

[b78] Patel R. K. & Jain M. NGS QC Toolkit: a toolkit for quality control of next generation sequencing data. PLoS ONE 7, e30619 (2012). 22312429 10.1371/journal.pone.0030619 PMC3270013

[b79] Krueger F. & Andrews S. R. Bismark: a flexible aligner and methylation caller for Bisulfite-Seq applications. Bioinformatics 27, 1571–1572 (2011). 21493656 10.1093/bioinformatics/btr167 PMC3102221

[b80] Garg R., Sahoo A., Tyagi A. K. & Jain M. Validation of internal control genes for quantitative gene expression studies in chickpea (*Cicer arietinum* L.). Biochem. Biophys. Res. Commun. 396, 283–288 (2010). 20399753 10.1016/j.bbrc.2010.04.079

[b81] Garg R., Bhattacharjee A. & Jain M. Genome-scale transcriptomic insights into molecular aspects of abiotic stress responses in chickpea. Plant Mol. Biol. Rep. 33, 388–400 (2015).

[b82] Jain M., Chevala V. V. S. N. & Garg R. Genome-wide discovery, analysis and implications of differential regulation of conserved and novel miRNAs in chickpea via deep sequencing. J. Exp. Bot. 65, 5945–5958 (2014). 25151616 10.1093/jxb/eru333 PMC4203128

[b83] Jain M., Nijhawan A., Tyagi A. K. & Khurana J. P. Validation of housekeeping genes as internal control for studying gene expression in rice by quantitative real-time PCR. Biochem. Biophys. Res. Commun. 345, 646–651 (2006). 16690022 10.1016/j.bbrc.2006.04.140

