# Supplementary material for: Divergent DNA methylation patterns associated with gene expression in rice cultivars with contrasting drought and salinity stress response
Source: Sci Rep. 2015 Oct 9;5:14922. doi: 10.1038/srep14922 (PMC4598828; doi:10.1038/srep14922)

**Supplementary information**

**Divergent DNA methylation patterns associated with gene expression in rice cultivars with contrasting drought and salinity stress response**

Rohini Garg^1^*, VVS Narayana Chevala^1^, Rama Shankar^1^ & Mukesh Jain^1^*

^1^Functional and Applied Genomics Laboratory, National Institute of Plant Genome Research (NIPGR), Aruna Asaf Ali Marg, New Delhi - 110067, India

**Supplementary Table S1** Details of bisulphite sequencing libraries generated in the study.

|  | **IR64** | **N22** | **Pokkali** |
| --- | --- | --- | --- |
| Total read pairs | 82237670 | 79798554 | 106014605 |
| **High-quality read pairs** | **82237654** | **79798533** | **106014601** |
| Uniquely mapped read pairs | 52632006 | 49374576 | 65592598 |
| Mapping efficiency (%) | 64.00 | 61.87 | 61.87 |
| **Genome coverage (%)** | **87.24** | **89.08** | **88.28** |
| Genome Cs | 163078985 | 163078985 | 163078985 |
| Cs covered | 131729131 | 136104482 | 134552064 |
| **C coverage** | **80.78%** | **83.46%** | **82.51%** |
| **Chloroplast DNA conversion rate (%)** | **99.55007** | **99.5742318** | **99.4819746** |
| Error rate | 0.0045 | 0.01 | 0.00755 |
| Total methylated Cs | 14906181 | 18210399 | 16819406 |
| **Total methylated C's (%)** | **11.32%** | **12.30%** | **11.50%** |

Only the reads mapped uniquely to the rice nuclear genome were included in the analysis to identify the methylated cytosines.

IR64, N22 and PK represent IR64, Nagina 22 and Pokkali rice cultivars, respectively.

**Supplementary Table S2** Summary of sequencing data generated for RNA-seq and mapping on the rice genome.

|  | **IR64** | **N22** | **PK** |
| --- | --- | --- | --- |
| Raw reads | 58565758 | 96289178 | 62150476 |
| High quality reads | 54230024 | 81129568 | 54962644 |
| Total mapped reads | 50117149 | 73756916 | 50848127 |

IR64, N22 and PK represent IR64, Nagina 22 and Pokkali rice cultivars, respectively.

**Supplementary Table S3** List of differentially expressed genes between the rice cultivars.

*Available as separate MS Excel file*

**Supplementary Table S4** List of genes with methylation level correlated with differential gene expression between the rice cultivars.

*Available as separate MS Excel file*

**Supplementary Table S5** Summary of small RNA sequencing data generated and pre-processing.

|  | **IR64** | **N22** | **Pokkali** |
| --- | --- | --- | --- |
| **Total raw reads** | 25959467 | 16051585 | 37240914 |
| **Unique reads after QC** | 1927142 | 1376538 | 2088005 |
| **Reads uniquely mapped to rice genome** | 1536225 (79.72%) | 1090668 (79.23%) | 1699184 (81.38%) |
| **Small RNA reads (21-24nt)** | 885816 (45.97%) | 653002 (47.44%) | 962624 (46.10%) |

IR64, N22 and PK represent IR64, Nagina 22 and Pokkali rice cultivars, respectively.

**Supplementary Table S6** List of primers used for real-time PCR analysis in this study.

| **Gene ID** | **Primer sequence** |
| --- | --- |
| LOC_Os09g07510 | CAGAGATGCTGCTGCAAACAA  ATCGCCCATTCTGGACCAA |
| LOC_Os11g31640 | TCCAGGGAGTGCCTTAGACAA  AGGTGTGGCAGGGAATGC |
| LOC_Os02g48560 | CATCCCGCCTCACTGCTT  CATGGACCACGTAGGAGAAGGA |
| LOC_Os03g22720 | TTCTTGGAGGCGTTGTCATG  AGGTTTTCACCTGGAGATTTGTG |
| LOC_Os10g39130 | CAACAAGATATCCAGCAAGTCAAAG  TGGACTCATCAAGAGCTTCAAGTTT |
| LOC_Os01g14610 | TCGTGGCGGAGGATGAGA  GGCCTCCATGCGAATGTT |
| LOC_Os02g42810 | GCCGAGAAAAGGGTTGCA  TGCATATCTCGAGCCCAAGTC |
| LOC_Os11g20160 | TGGTGACGCGTTCCAGTGTA  GCAGAACAAGCTTGAGCATGACT |
| LOC_Os08g01794 | AGGGAATGATCGACACAAAAATG  TTTGCGGCGTGTAACTTGAC |
| LOC_Os03g48970 | GAGAAAGCCGAACCAAACAAAG  GGAGGGATCCGGCGATAC |
| LOC_Os11g01154 | CTCAGGATGCTCTGCGACTTC  TAGCGCCATTCTGGACAAGA |
| LOC_Os06g43860 | GGCTCACTCTAAATGGCCATACC  AACCCTGTTTCCTGCACCAA |
| LOC_Os04g16722 | GGAGCACCTAACAACGCATCTT  CCCCAGAATGAAAGGGTGATC |
| LOC_Os01g72530 | GGCTCCGACCAGGACATC  TGAGATCGAACCTGCAGATCA |
| LOC_Os01g72370 | CCTACTACTACTGGTCTGGCTTGGT  CCCGCTTCCGTCTCAAAAG |
| LOC_Os01g53220 | TCCTCCTCCCCTCCTACTTCA  GCGGAATCCGTAGGTGTTGA |

**Supplementary Figure S1** DNA methylation level in each sequence context (CG, CHG and CHH) in three rice cultivars. The fraction of mCs with different levels of methylation are shown in the line graph. The average value of methylation level in each sequence context is also shown.

**Supplementary Figure S2** Global methylome maps of rice in different rice cultivars. Density plots of methylcytosines in different sequence contexts (I, CG; II, CHG and III, CHH), genes (IV), transposable elements (V) and small RNAs (VI) are shown on both DNA strands (outside, sense strand and inside, antisense strand). Chromosome name and scale are indicated on the outer rim.

**Supplementary Figure S3** Box-plot showing distribution of DNA methylation level on both DNA strands in each sequence context in three rice cultivars.

**Supplementary Figure S4** Fraction of mCs identified in different genomic features in each sequence context in three rice cultivars.

**Supplementary Figure S5** DNA methylation patterns in the gene body and flanking sequences in different rice cultivars. Average methylation level of mCs within gene body and flanking (upstream and downstream) sequences in each sequence context in different cultivars. TSS, transcriptional start site and TTS, transcriptional termination site.

**Supplementary Figure S6** Fraction of differentially methylated regions (DMRs) identified within the gene body or flanking (2 kb upstream and downstream) sequences among rice cultivars.

**Supplementary Figure S7** Gene ontology (GO) categories represented in the DMR-associated genes for N22/IR64 and PK/IR64.

**Supplementary Figure S8** DMRs associated with on-off gene expression state. The scatter plots show correlation of DMRs in N22/IR64 (a) and PK/IR64 (b) with switch on-off expression state of nearby genes (within 2 kb). The plots show that hypermethylation leads to switch-off state of gene expression and hypomethylation leads to switch-on state of gene expression. (c) GO enrichment analysis of DMR-associated genes showing on-off gene expression state. The significantly enriched GO terms are highlighted in different colours as per scale (P-value) given at the bottom.


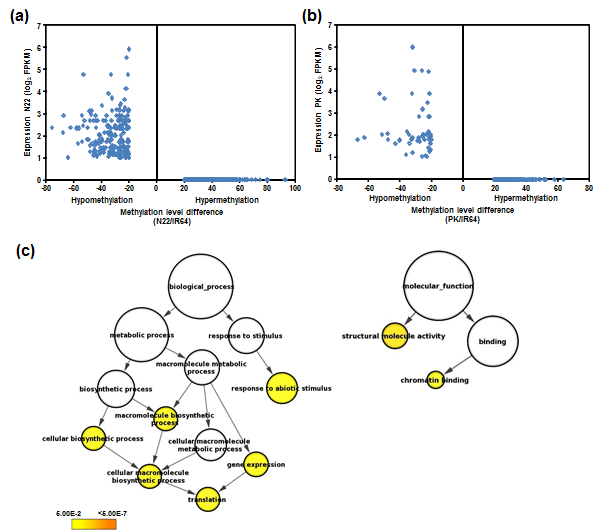


**Supplementary Figure S9** Validation of DNA methylation results. Scatter plot showing correlation of methylation levels predicted by whole genome bisulphite sequencing (WGBS) and locus-specific bisulphite sequencing (LSBS) in IR64 (a), Nagina 22 (b) and Pokkali (c) rice cultivars. The Pearson correlation (r) obtained for each sequence context has been indicated.

**Supplementary Figure S10** Validation of differential gene expression results. Scatter plot showing correlation of transcript levels (log2 fold change) predicted by RNA-seq and qRT-PCR. The Pearson correlation (r) has been indicated.


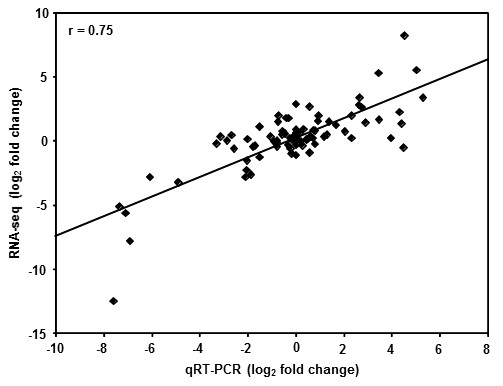

Supplement: Supplementary Information [file srep14922-s1.doc]
